# Testing the genomic stability of the Brazilian yellow fever vaccine strain using next-generation sequencing data

**DOI:** 10.1098/rsfs.2020.0063

**Published:** 2021-06-11

**Authors:** Amanda Araújo Serrão de Andrade, André E. R. Soares, Luiz Gonzaga Paula de Almeida, Luciane Prioli Ciapina, Cristiane Pinheiro Pestana, Carolina Lessa Aquino, Marco Alberto Medeiros, Ana Tereza Ribeiro de Vasconcelos

**Affiliations:** ^1^ National Laboratory for Scientific Computing, Bioinformatics Laboratory (LABINFO), Avenida Getúlio Vargas, 333, Quitandinha 25651-075, Petrópolis, Rio de Janeiro, Brazil; ^2^ Fiocruz, Bio-Manguinhos, Recombinant Technology Laboratory (LATER), Brazilian Ministry of Health, Rio de Janeiro, Brazil

**Keywords:** yellow fever vaccine, 17DD, genetic stability, genetic diversity, attenuated viral vaccines

## Abstract

The live attenuated yellow fever (YF) vaccine was developed in the 1930s. Currently, the 17D and 17DD attenuated substrains are used for vaccine production. The 17D strain is used for vaccine production by several countries, while the 17DD strain is used exclusively in Brazil. The cell passages carried out through the seed-lot system of vaccine production influence the presence of quasispecies causing changes in the stability and immunogenicity of attenuated genotypes by increasing attenuation or virulence. Using next-generation sequencing, we carried out genomic characterization and genetic diversity analysis between vaccine lots of the Brazilian YF vaccine, produced by BioManguinhos–Fiocruz, and used during 11 years of vaccination in Brazil. We present 20 assembled and annotated genomes from the Brazilian 17DD vaccine strain, eight single nucleotide polymorphisms and the quasispecies spectrum reconstruction for the 17DD vaccine, through a pipeline here introduced. The V2IDA pipeline provided a relationship between low genetic diversity, maintained through the seed lot system, and the confirmation of genetic stability of lots of the Brazilian vaccine against YF. Our study sets precedents for use of V2IDA in genetic diversity analysis and *in silico* stability investigation of attenuated viral vaccines, facilitating genetic surveillance during the vaccine production process.

## 1. Background

Yellow fever virus (YFV) causes an acute febrile disease affecting humans and non-human primates called yellow fever (YF) [1]. YF is endemic to the tropical areas in 31 African and 10 Latin American countries [2]. YF outbreaks affected Paraguay and Argentina (2007–2009), Uganda (2010), Sudan and Ethiopia (2012–2013) and recently southern Brazil (2016–2019) [2,3]. These outbreaks led to the intensification of vaccination campaigns and repeatedly depleted the vaccine stocks in both continents [4].

All YF vaccines used today are based on an attenuated YFV, derived from a clinical isolate (Asibi strain) and attenuated by serial passaging [5]. WHO-prequalified YF vaccines belong to either of the two main substrains of the original attenuated YFV: 17D-204 at passage number 204 and 17DD at passage number 195 [6]. While the live attenuated 17D-204 vaccine is manufactured in the USA, France, China, Senegal and Russia [7] and used worldwide [6], the 17DD substrain is produced and distributed exclusively in Brazil by BioManguinhos (Oswaldo Cruz Foundation, Fiocruz), which is linked to the Brazilian Ministry of Health. It also supplies the YF 17DD vaccine to other countries in South America and Africa [8].

The 17DD vaccine is produced in specific pathogen-free chicken embryos and a seed lot system has been used since 1941 to ensure genetic stability and safety of the vaccine lots [9]. In this system, a working seed lot is produced to give rise to new vaccine lots with the same number of cell passages, generating the necessary standardization for this productive process. For several years, the 17DD vaccine secondary seed lot 102/84 was used to produce millions of vaccine doses until it derived a new working seed, the 993FB013Z [10]. This new seed lot was analysed regarding the *in vivo* genomic stability following established protocols and is currently used to produce new vaccine lots at one passage level [11].

Genomic characterization of attenuated viruses as well as genetic diversity analysis between vaccine lots is extremely important for vaccine quality control, investigation of genetic stability and maintenance of the attenuated phenotype [12]. Both vaccine strains against YF are not biological clones but consist of viral populations with some level of genetic diversity maintained through the seed lot system of vaccine production [10].

The genetic diversity between viral genomes is mainly caused by insertions of single nucleotide polymorphisms (SNPs), recombination, or reordering (of fragmented viral genomes) by the replicase enzymes responsible for viral replication. The rate of intrinsic error of the replicase enzymes determines the mutation rate for each viral species and the range of genetic variation, in which natural selection can act [13]. Natural populations of most RNA viruses, including YFV, may have different viral quasispecies generated by the occurrence of different SNPs. Viral quasispecies are a group of interactive variants, often referred to as sub-populations [14].

The presence of SNPs and quasispecies in viral vaccine stocks negatively influences genetic stability. Phenotypic changes as a result of high genetic diversity can potentially impact immunogenicity (by increasing attenuation or virulence) and affect the safety profile of live attenuated viral vaccines [15]. Therefore, low genetic diversity and low phenotypic changes are required to ensure the genetic stability of viral vaccine stocks. In a stable and safe vaccine stock, a phenotype should not accumulate mutations beyond the level present in past vaccine stocks with good clinical records [16]. Genetic stability testing involves the monitoring of genetic diversity and is a fundamental step in confirming the safety of an attenuated viral vaccine [17].

Although previous studies have used Sanger sequencing to identify genetic diversity in YFV 17DD and 17D vaccine stocks [9–11,18], this sequencing technique shows limitations regarding the detection of low frequency and co-occurred SNPs. The limitations of Sanger sequencing may be overcome by next-generation sequencing (NGS), which generates the required depth of coverage for the analysis of the variants in viral populations within a sample. It allows for high-throughput detection of a vast amount of SNPs and their co-occurrences in a genome [19].

Detection of genetic diversity from raw sequencing data is a multistep task and can be executed using numerous tools and resources. To accurately extract relevant information from NGS data it is crucial to choose reliable tools, fine-tune them and correctly interpret their results [19]. Previous studies have used NGS directly on viral vaccine stocks [15,20,21] and applied different methods to infer genetic diversity. The main limitations of previous NGS studies were: the lack of a simple automated pipeline, methodological standardization and quasispecies reconstruction analysis. Requiring user input during each step of the process, and the lack of a reproducible computational pipeline may provide slower and incorrect results [19,22], and reproducibility issues [19,23]. Not performing quasispecies reconstruction is an important limitation since identifying the occurrence and co-occurrence of nucleotide-level mutations is more informative than focusing solely on the dominant viral phenotypes. The reconstruction of possibly mutated phenotypes allows the prediction of correct genetic stability in attenuated viral vaccine stocks [13,24].

In this study, we aim to sequence, assemble and annotate the viral genomes of 17DD vaccine stocks, while inferring their genetic diversity, and testing their overall genetic stability. For this purpose, we developed a bioinformatic pipeline specific to handle NGS data from viral vaccine stocks. This pipeline allows for reproducible results and provides a fast, accurate, *in silico* solution to identify genomic diversity in 17DD vaccine lots based on Illumina shotgun sequencing data.

## 2. Methods

### 2.1. Samples

BioManguinhos (Fiocruz) provided 20 vaccine samples (table 1), including the primary (458 IOC), secondary (102/84) and current working seed (993FB013Z) lots used in the manufacture of vaccines, and 17 vaccine lots produced from 2007 to 2018.

**Table 1.** List of all vaccine lots sequenced for this study.

sample ID	description	production year
458 IOC	primary seed lot	1973
102/84	secondary seed lot	1984
993FB013Z	working seed lot	1999
005FB003Z	vaccine lot derived from 102/84	2000
6Z	vaccine lot derived from 993FB013Z	2007
36Z	vaccine lot derived from 993FB013Z	2007
23Z	vaccine lot derived from 993FB013Z	2010
24Z	vaccine lot derived from 993FB013Z	2010
56Z	vaccine lot derived from 993FB013Z	2012
59Z	vaccine lot derived from 993FB013Z	2012
72Z	vaccine lot derived from 993FB013Z	2012
7Z	vaccine lot derived from 993FB013Z	2015
8Z	vaccine lot derived from 993FB013Z	2015
9Z	vaccine lot derived from 993FB013Z	2015
1Z	vaccine lot derived from 993FB013Z	2016
2Z	vaccine lot derived from 993FB013Z	2016
1Z	vaccine lot derived from 993FB013Z	2018
2Z	vaccine lot derived from 993FB013Z	2018
3Z	vaccine lot derived from 993FB013Z	2018
17Z	vaccine lot derived from 993FB013Z	2018

**Figure 1.**
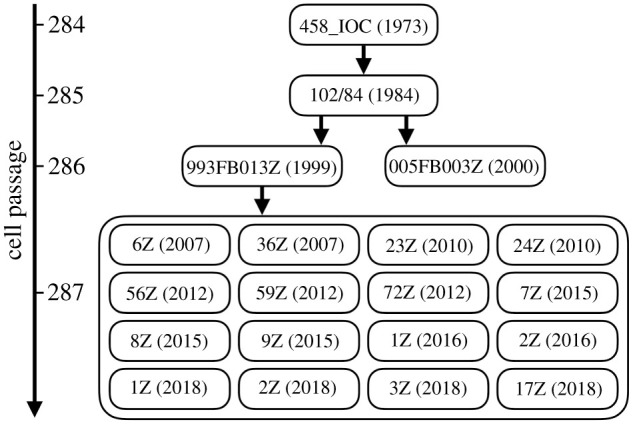
The YF vaccine 17DD seed lots are used for production in Brazil by BioManguinhos. Each lot code is composed of its name followed by the year of production in parentheses. The vertical arrow on the left indicates the number of cell passages from the original virulent strain Asibi. The 458_IOC primary seed lot was used to prepare the secondary seed lot 102/84. This seed yielded the YF vaccine from 1984 to 2002, when the vaccine batch 993FB013Z was turned into the working seed. Vaccine production from the 993FB013Z seed strain has been ongoing since 2002 and the vaccine virus is currently at passage level 287 as of 2020 [10].

For easier identification, we assigned new sample IDs composed of the vaccine lot name followed by the year of production in parentheses. Figure 1 shows the parental relationship, established through the seed-lot vaccine production system, between each sample and the history of YF vaccine production in Brazil, from 1973 to 2018.

### 2.2. RNA extraction, cDNA synthesis and PCR amplification

RNA extraction, primer design, amplification and the construction of amplicon libraries for 20 samples were carried out in BioManguinhos, according to the protocols described in [11]. Amplicons libraries were diluted and subsequently pooled to equivalent molar ratios.

### 2.3. Sequencing and pre-processing

Library sequencing was done on an Illumina MiSeq instrument, with Nano Kit v.2 2 × 250 bp paired-end chemistry. PhiX control oligonucleotides were spiked into the run (10%) to add base diversity. Sequencing quality was evaluated using FastQC (http://www.bioinformatics.bbsrc.ac.uk/projects/fastqc) to remove low-quality bases (*Q* = <30). Adapter sequences were removed with Trimmomatic v.0.39 [25].

### 2.4. Genome assembly, consensus sequence and functional annotation

We processed the raw NGS data files for each vaccine lot separately. *De novo* genome assembly was performed using SPADES v.3.11.1 [26], with default parameters. A consensus sequence was created for each assembled vaccine lot. A minimum Phred score of 30 and 100 bp length were required to use a sequencing read for genome assembly. The resulting genomes had a minimum coverage of 100×.

The functional annotation was performed using Geneious 7.0 (https://www.geneious.com) based on patterns and annotations from the YFV genome present in the public databases (National Center for Biotechnology Information (NCBI) and the Ebola and Hemorrhagic Fever Viruses Database (HFV) from Los Alamos National Laboratory (LANL)).

### 2.5. Normalization and downsampling

To make sure the total number of reads in each sequenced lot is equal and directly comparable, the total read count normalization by the scaling factor method [27] was applied by using a custom script in R v.3.4.4. This method standardizes the data between samples by calculation of scale factor according to the total read count in a given sample to a common value across all vaccine lots and accounts exclusively for the differences in sequencing depth, and no other sources of variability. Downsampling of vaccine lots was performed using Picard Tools DownsampleSam v.1.107 (http://picard.sourceforge.net/) with default parameters, in which subsets of reads of each sample were randomly selected to proceed to the next steps.

### 2.6. Viral vaccine genetic diversity analyser (V2IDA) pipeline

**Figure 2.**
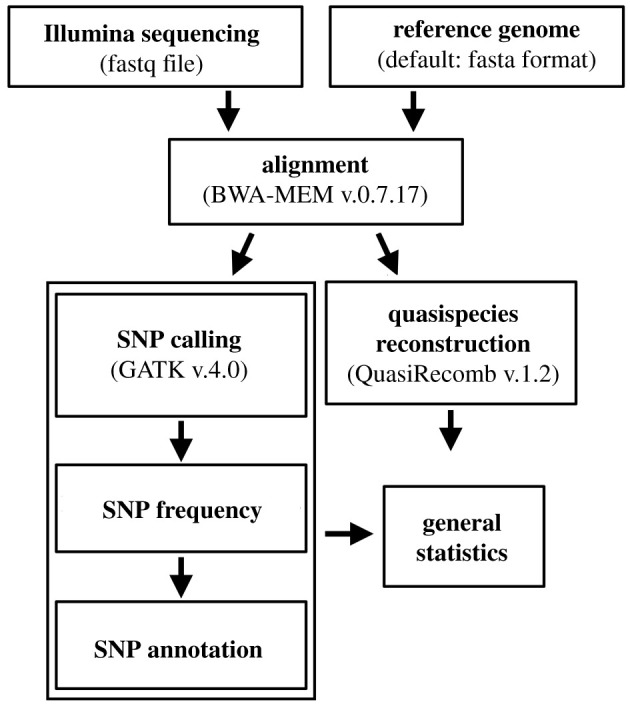
V2IDA pipeline. Available on GitHub (github.com/aandradebio/V2IDA).

Upon obtaining a reference consensus genome we used the V2IDA pipeline to process the sequencing data and obtain all subsequent results. V2IDA runs each vaccine lot independently, requires Illumina shotgun sequencing data, and a reference consensus genome as input. V2IDA performs the viral genetic diversity analysis straight from the raw sequence data, aligning the reads to a reference genome, followed by SNP calling, and quasispecies reconstruction (figure 2). Once the pipeline is finished, it generates multiple files comprising the general statistics for every analytic step in a manner compatible to be opened by Web browsers or text editors. The code necessary to run the V2IDA pipeline is freely available on GitHub (github.com/aandradebio/V2IDA).

### 2.7. Alignment

Reads were aligned to a reference genome (993FB013Z, assembled in this study) using BWA-MEM v.0.7.17 [28] with default parameters. The BAM file was created using Samtools [29]. PCR duplicates were removed with MarkDuplicate tool v.1.107 and the BAM file was sorted and indexed using Picard Tools v.2.18 (http://picard.sourceforge.net/).

### 2.8. Single nucleotide polymorphism calling

SNPs and insertion and deletions (INDELS) were called using the Genome Analysis Toolkit (GATK v.4.0) HaplotypeCaller tool [30]. Best practices steps to call genetic variants such as creating realignment targets, base quality score recalibration (BQSR) and variant quality score recalibration (VQSR) were used to increase the analysis specificity according to the GATK recommendations. While the BQSR tool recalibrated base quality scores by applying an error probability model to the bases, the VQSR tool used machine learning methods to estimate the relationship between the SNPs called and the probability which an SNP is a true genetic variant, rather than a sequencing or data-processing artefact.

Hard-filtering was applied to select SNPs based on base confidence (Depth (DP) > 10.0, Quality (QUAL) > 500, QualByDepth (QD) < 2.0 and Mapping Quality (MQ) > 40.0) and based on the possibility of strand bias by performing a Phred-scaled *p*-value using Fisher's exact test (FisherStrand (FS) < 60.0) and Symmetric Odds Ratio Test ((SOR) > 4.0). Finally, the output was a variant calling file (VCF) per sample containing SNP frequencies and annotations. SnpEff build tool was used to build the custom database from GFF files of YFV complete genomes found in the NCBI and HFV public databases. The same YFV genomes were previously used to perform functional annotation of the 17DD genomes.

### 2.9. Quasispecies reconstruction

We used the QuasiRecomb (v.1.2) algorithm [31], which employs a probabilistic model based on Jumping Hidden Markov Model to infer viral quasispecies from deep-coverage NGS data, using an expectation-maximization algorithm for maximum *a posteriori* parameter estimation. Even though QuasiRecomb is adapted to accept global read alignments in BAM format, the whole genome was subdivided into five regions containing up to 2000 nucleotides and one region of 1000 nucleotides. According to previous studies and software recommendations, this strategy helps increase software accuracy and decrease false-positive results [32,33]. QuasiRecomb ran on the selected genomic region, ignoring any gaps (-noGaps) and without allowing recombination (-noRecomb). The computational algorithm produces a list of reconstructed quasispecies and their frequencies of occurrence in each region. All reconstructed quasispecies with a total frequency below 1% were excluded to differentiate true SNPs and SNPs caused by sequencing errors [32].

### 2.10. Phylogeny analysis of the reconstructed quasispecies

The output files from the quasispecies reconstruction were concatenated for each region, aligned, and used for phylogenetic analysis, using the neighbour-joining method with the Jukes-CantorBioNJ evolutionary model and 1000 bootstrap replicates, as implemented in Seaview v.4.7 [34]. Basic statistical analyses (arithmetic mean, median and standard deviation) were performed using custom scripts in R v.3.4.4.

## 3. Results

### 3.1. Sequencing, assembly and annotation

We sequenced, assembled and annotated the complete genome of 20 samples from different cell passage levels of the 17DD vaccine strain. NGS generated a total of 183 million reads, with an average Phred quality score of 36. Only reads with a minimum size of 200 and a maximum of 250 nucleotides (nt) were selected. All the data generated were used for the subsequent analyses.

The *de novo* assembly of the 20 vaccine lot complete genomes used from 87.71% to 98.51% of the total generated reads. The 20 assembled genomes were used in the next step for functional annotation. The comparison of all 17DD vaccine lots revealed low genetic variation, with an average nucleotide identity of 99.8% and amino acid identity values ranging from 99.9% to 100%. All 20 vaccine lots sequenced for the 17DD strain had identical consensus sequences and annotation.

The complete genome of 17DD vaccine strains is 10 862 nt long, coding a large polyprotein of 10 523 nucleotides processed into the viral structural proteins: capsid (C), pre membrane (PrM), membrane (M), envelope (E) and the viral nonstructural proteins: NS1, NS2A, NS2B, NS3, NS4A, NS4B, NS5. In addition to proteins, the viral genome has untranslated regions (UTR) at the 5′ and 3′ ends (table 2).

**Table 2.** The functional annotation for the YF 17DD strain. UTR, untranslated region; C, capsid; prM, pre membrane; M, membrane; E, envelope; NS, nonstructural proteins.

genomic region	type	initial nucleotide	final nucleotide
5′UTR	untranslated region	1	118
Polyprotein	translated region	119	10523
C	mature peptide	119	504
prM	mature peptide	505	1135
M	mature peptide	860	1135
E	mature peptide	1136	2701
NS1	mature peptide	2702	3951
NS2A	mature peptide	3952	4437
NS2B	mature peptide	4438	4797
NS3	mature peptide	4798	6690
NS4A	mature peptide	6691	7545
NS4B	mature peptide	7546	7903
NS5	mature peptide	7904	10523
3′UTR	untranslated region	10524	10862

### 3.2. Alignment and single nucleotide polymorphism calling

Given the identical consensus sequence and functional annotation for all the 20 vaccine lots, the annotated consensus genome of the working seed lot 993FB013Z was used as the reference genome for alignment due to its parental relationships with other vaccine samples. By using the V2IDA pipeline, the raw data of each sequenced vaccine lot were aligned to the 993FB013Z genome. After genome alignment and before SNP calling we performed normalization and downsampling, so differences between depth coverages do not interfere with SNP calling and comparisons of the genetic diversity profiles between vaccine lots. Electronic supplementary material, table S1, contains genome assembly and alignment statistics.

**Figure 3.**
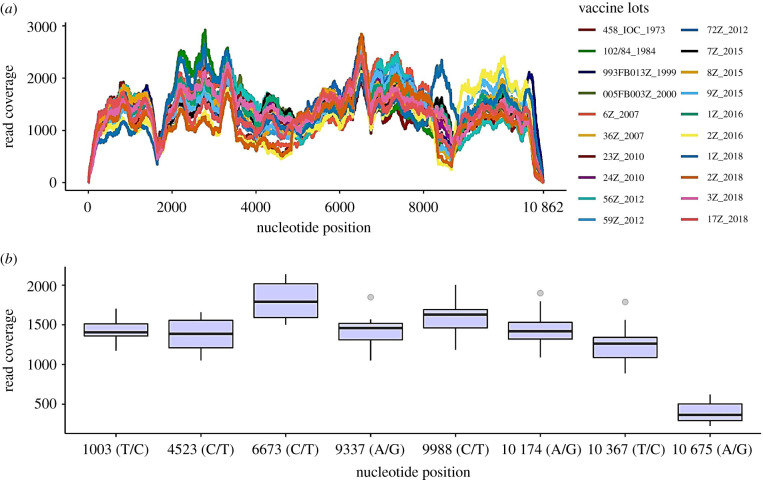
Whole-genome and variant base read coverage. (*a*) Multiple line plots showing whole-genome read coverage for all 20 sequenced vaccine lots. (*b*) Boxplot for median read coverage at variant nucleotide positions. Horizontal bars indicate median values, boxes depict the first and third quartiles, whiskers depict the minimum and maximum values, and outliers are found as points. SNPs are characterized by the nucleotide position and the canonical base followed by the variant base.

All vaccine lots presented similar read coverage patterns across the genome after downsampling (figure 3*a*), with average read coverage ranging from 1175× to 1427× (electronic supplementary material, table S1). We detected eight highly covered SNPs in the 17DD vaccine lots (figure 3*b*). The SNPs 6673 (C/T) and 10 675 (A/G) showed the highest (2138×) and the lowest read coverage (224×), respectively. Electronic supplementary material, table S2, contains the SNP frequencies and SNP base coverage per vaccine lot.

**Figure 4.**
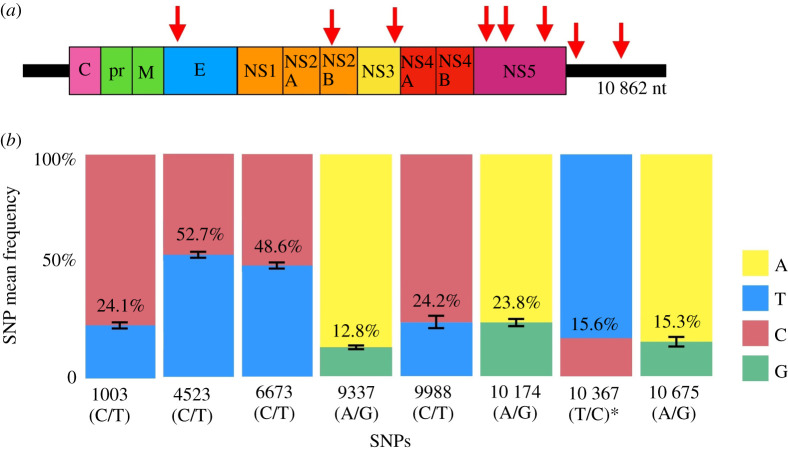
Genomic location and frequency of the eight identified SNPs. (*a*) Red arrows indicate the genomic position of the found SNPs and the corresponding coding genes (C, capsid; prM, pre membrane; E, envelope; NS1–NS5, nonstructural proteins). (*b*) Each block represents a variant and its nucleotide position; the percentage data represent the variant mean frequency (obtained from the arithmetic mean frequency of the variant in each of the 20 samples used in the analysis) and the colours correspond to nucleotide bases. *Variant found only in sample 993FB013Z13Z.

The eight SNPs detected were found in the envelope protein (1), NS2B protein (1), NS3 protein (1), NS5 protein (3) and 3′UTR (2) of the viral genome (figure 4*a*). All SNPs are transition-type substitutions and do not code for changes in the amino acid sequences. All 20 analysed vaccine lots presented the same SNP profile, except for the working seed lot 993FB013Z that showed an additional SNP at position 10 367 (with 15.6% frequency) located in the 3′UTR. SNP average frequencies varied from 12.8% to 52.7%. The highest frequency was observed for SNP at position 4523 (C/T), located in the NS2B protein, and the lowest frequency for SNP at position 9337 (A/G), located in the NS5 protein (figure 4*b*).

### 3.3. Quasispecies reconstruction

Quasispecies reconstruction and their frequency estimation analysis based on whole-genome sequences were performed on six distinct regions of the viral genome, namely: (i) region one (0–1000 nt), (ii) region two (1000–3000 nt), (iii) region three (3000–5000 nt), (iv) region four (5000–7000 nt), (v) region five (7000–9000 nt) and (vi) region six (9000–10 863 nt) (electronic supplementary material, table S3). The SNP calling performed independently by the QuasiRecomb (v.1.2) algorithm has detected the same SNPs as the previous analysis using GATK (4.0) HaplotypeCaller tool. Therefore, all the SNPs were confirmed at the same position in each region of the reconstructed quasispecies.

**Figure 5.**
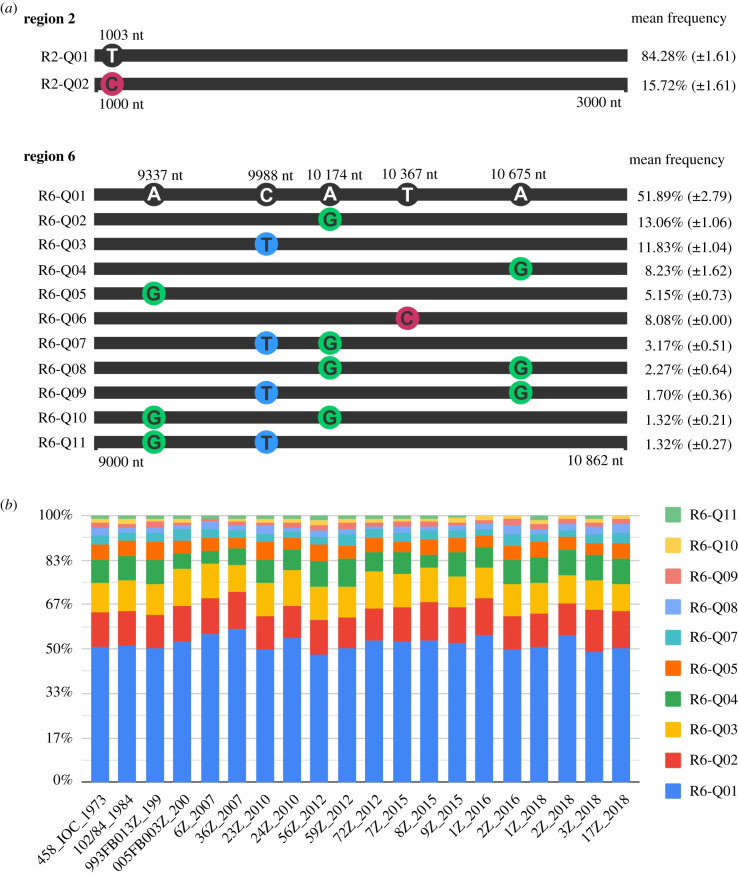
Quasispecies reconstructed for the genomic region two (1000–3000 nt) and region six (9000–10 862 nt). (*a*) Representation of the nucleotide variation between reconstructed quasispecies for regions two and six of the viral genome. The nucleotides with a black circle around are the canonical nucleotides, while nucleotides with a coloured circle are SNPs. Each quasispecies population was assigned a code, to the left, and the mean frequency of the indicated population with standard deviation, to the right. (*b*) Frequency distribution per reconstructed quasispecies among each vaccine lot.

Region one (0–1000 nt) presented no SNPs and the canonical quasispecies as the only reconstructed quasispecies with 100% frequency. This region comprises the capsid, pre membrane and part of the membrane proteins. The genomic region two (1000–3000 nt) codes the envelope protein, and showed the SNP 1003 (T/C), the canonical quasispecies reconstruction (frequency of 84.28%), and a new quasispecies reconstruction with 15.72% frequency (figure 5*a*).

Region three (3000–5000 nt) reported the SNP 4523 (C/T) and region four (5000–7000 nt) the SNP 6673 (C/T). Two quasispecies were reconstructed for each region. The SNPs presented a 50% frequency for both alternative nucleotides in all vaccine lots, resulting in the absence of a consensus nucleotide for these genomic positions. Region three codes the NS1, NS2A, NS2B and part of the NS3 protein, while region four codes part of the NS3 and part of the NS4A viral protein.

Region five (7000–9000 nt) reported no SNP and only the canonical quasispecies was reconstructed with 100% frequency. This region codes part of the NS4A and the full NS4B proteins. Contrasting with region five, region six (9000–10 862 nt) was the highest variable region showing five covariate SNPs and 11 quasispecies reconstructed (figure 5*a*). This region comprises the NS5 protein and the 3′UTR. Figure 5*b* shows the frequency distribution of reconstructed quasispecies for all vaccine lots in region six. Similar to other regions, the canonical quasispecies (R6-Q01) had the highest frequency in every lot.

### 3.4. V2IDA pipeline

The V2IDA pipeline was used to analyse the 17DD strain genetic diversity. To choose the algorithms enrolled in the V2IDA pipeline, nine state-of-the-art algorithms (three for each step) were previously selected after careful literature revision (electronic supplementary material, table S4) from previous experiments [19,22,23,32,33,35,36,38,39]. The chosen algorithms have the same fundamental characteristics: open-source, executable from the command line and widely used in the scientific community (including in studies with viral RNA genomes). The use of these algorithms allows future studies to have access to them, facilitating reproducibility.

For the genetic diversity experiments, we tested the sensitivity and specificity metrics. During the initial tests, sensitivity was prioritized over specificity to minimize the risk of false negatives. Therefore, GATK v.4.0 was run without applying any filtering criteria and a total of 12 SNPs were found scattered across the genome. After applying hard-filters, a total of eight SNPs remained (detailed in §3.2). The use of hard-filtering increased specificity by detecting true SNPs and removing false-positive SNPs. The hard-filtering tuning was selected based on data (e.g. read length, read coverage and quality scores) and based on the 17DD genome (e.g. genome size and absence of recombination events).

## 4. Discussion

In this study, we combined high-depth NGS data and *in silico* analyses to investigate the genetic diversity of 20 vaccine lots of the 17DD strain and assess vaccine stability. The 17DD vaccine strain included primary, secondary, working seed lot and vaccine lots used in the past 12 years of vaccination in Brazil. In addition to the analysis of SNP occurrence and SNP co-occurrence patterns, we were able to provide fully annotated genomes from the Brazilian YFV vaccine.

By investigating the YF vaccine genetic diversity at the nucleotide level, we have identified eight high-frequency synonymous SNPs. The SNPs at positions 1003 (T/C), 4523 (C/T), 6673 (C/T), 9337 (A/G), 9988 (C/T), 10 174 (A/G) and 10 675 (A/G) were previously described in the scientific literature [9–11], without information about frequency rates and SNP co-occurrence. The SNP at position 10 367 (T/C) was firstly identified in this paper, likely due to limitations of previous sequencing technologies.

To date, there are no reports of genomic characterization or analysis of genetic diversity among vaccine lots of 17DD strains using NGS data. In previous studies [9–11], Sanger sequencing was the method of choice for analysing 17DD vaccine lots. However, this method only allows determining the consensus sequence of the virus population. The consensus sequence only aggregates the nucleotides with the highest frequency within the sequenced sample. The limitations of the previous studies were overcome in the present study since the use of NGS allowed the characterization and proper quantification of the SNP profile found with a minimum frequency of 12.8%, not detected only by analysing the consensus sequence. The high-depth detection of SNPs was possible due to the high read coverages (average read coverage ranged from 1175× to 1427×) attained using NGS.

The machine learning algorithm, statistical tests and hard-filtration criteria used from GATK v.4.0, and implemented in the V2IDA pipeline, increased the genetic diversity analysis specificity. Therefore, all SNPs detected in this study are less likely to be associated with sequencing errors, poor variant detection and misalignment to the genomic reference [39].

Our genetic diversity analysis indicates that the most variable region of the YFV genome is contained towards the 3′ end of the genome, including the NS5 protein and 3′UTR in the reconstructed region six (9000–10 862 nt) with eleven quasispecies reconstructed for five co-occurred SNPs, followed by the reconstructed regions: three (3000–5000 nt), four (5000–7000 nt) and one (1000–3000 nt), with one SNP and two quasispecies each. This SNP co-occurrence pattern was expected, given the findings of previous genetic diversity studies in 17DD [11] and 17D [21,40] vaccine strains which identified higher genetic diversity mainly in the 3′-UTR and NS5, NS3, NS2A, NS2B, NS4A, NS4B and E proteins.

Despite the coexistence of a small number of different viral quasispecies within a 17DD vaccine lot, all 20 vaccine lots presented the same consensus sequence, amino acid sequences and functional annotation. This result is in accordance with previous studies [7,11,21,41] in which the authors concluded that the parental strains were found to consist of diverse quasispecies, while the attenuated YFV had very little genetic diversity. Therefore, live attenuated RNA virus vaccines should display a highly stable consensus sequence and a restricted SNP profile and quasispecies composition rather than the parental strains, as a consequence of the attenuation process.

The Brazilian YFV vaccine lots are highly genetically homogeneous and stable with eight synonymous SNPs. Seven out of eight SNPs were detected in all 20 analysed vaccine lots, which suggests its stable propagation through cell passages from lot to lot during the seed lot system of vaccine production. According to previous reports [15,41], the successive cell passages from the seed-lot system lead to the achievement of a high-fidelity replication complex that does not accumulate SNPs as the replication complex from wild-type YFV.

Our results confirm the 17DD strain genetic stability and the high efficiency of the seed lot system, implemented in BioManguinhos, to maintain the genetic stability of attenuated viruses. The genetic stability of the 17DD strain may largely lower the risks for antigenic drift or evolution of revertant virus vaccines [15,21,40], and may explain the excellent safety history for this vaccine.

Highlighting the importance of our study, we reinforce the monitoring of genetic diversity and *in silico* genetic stability testing as part of the vaccine manufacturing process to ensure the safety of all vaccine lots administered to the population. However, existing pipelines that analyse viral NGS samples do not accurately extract genetic diversity information when dealing with viral vaccine samples due to the lack of specific parameters [22,38], use of inappropriate tools [19,23,36], and not performing quasispecies reconstruction [15,35,37,38]. This often leads to false results and affects negatively the sensitivity and specificity metrics.

In this context, we have developed a computational pipeline called V2IDA to investigate vaccine stability through genetic diversity analysis of viral vaccine lots using NGS data. The V2IDA pipeline was designed for non-bioinformatician users and automates the steps required for viral genome diversity analysis. The approach introduced here was created to have high sensitivity and specificity in identifying SNPs and reconstructing quasispecies for 17DD viral vaccines, given the limitations of available algorithms and the absence of a gold standard methodology.

The use of bioinformatic tools, such as the V2IDA pipeline, may speed up the detection of reversion to virulence, decrease the number of post-vaccine adverse reactions and decrease the precedents for the use of animal models and laborious laboratory tests. Thus, future studies should focus on testing different parameters and benchmarks, in the search of a gold standard testing procedure of vaccine lots.

## 5. Conclusion

We fully assembled and annotated 20 vaccine lots from multiple cell passages of the 17DD strain, used for the production of the Brazilian YF vaccine. Our genetic diversity results provided invaluable insights into the viability and stability of the 17DD vaccine strain. The 17DD genetic stability may be linked to the seed-lot process of vaccine production performed by BioManguinhos and the achievement of a high-fidelity replication complex in the attenuated YFV genotype.

The V2IDA pipeline, introduced here, was used to establish the relationship among genetic diversity, vaccine stability and the possible reversion to virulence caused by the presence of SNPs and quasispecies in 17DD vaccine lots. V2IDA was developed to have the high sensitivity and specificity, being capable of taking NGS data as input and providing genetic diversity analyses and quasispecies reconstruction.

We emphasize the importance of testing the genomic stability of vaccine strains as an important part of quality control during vaccine manufacturing, and we suggest the use of V2IDA to automate and facilitate reproducibility in the genetic surveillance procedures, ensuring the safety profile of the vaccines administered to the population.

## Data accessibility

The sequences for all 20 vaccine lots were deposited in the NCBI Bioproject database under accession number PRJNA592122. The annotated consensus sequence of 102-84 (1984) vaccine seed lot was submitted to the NCBI Genbank database under the accession number MN737509. V2IDA pipeline is available on GitHub.com (github.com/aandradebio/V2IDA). The extra datasets supporting this article have been uploaded as part of the electronic supplementary material (table S1: genome assembly and alignment statistics; table S2: SNP statistics; table S3: quasispecies reconstruction statistics; table S4: comparison of algorithms used to develop theV2IDApipeline).

## References

[1] Monath TP, Gershman M, Erin Staples J, Barrett ADT. 2012 Yellow fever vaccine. In Vaccines, 6th edn, pp. 870-968. Amsterdam, The Netherlands: Elsevier Inc.

[2] Monath TP, Vasconcelos PFC. 2015 Yellow fever. J. Clin. Virol. **64**, 160-173. (10.1016/j.jcv.2014.08.030). 25453327

[3] Calado AFS, Paz FAN. 2020 Análise de perfil epidemiológico e incidência de febre amarela no Brasil. Res. Soc. Dev. **9**, 9932271. (10.33448/rsd-v9i3.2271)

[4] Vannice K, Wilder-Smith A, Hombach J. 2018 Fractional-dose yellow fever vaccination: advancing the evidence base. N. Engl. J. Med. **379**, 603-605. (10.1056/nejmp1803433) 29995585

[5] Theiler M, Smith HH. 1937 The use of yellow fever virus modified by in vitro cultivation for human immunization. J. Exp. Med. **65**, 787-800. (10.1084/jem.65.6.787) 19870634 PMC2133527

[6] Stock NK, Boschetti N, Herzog C, Appelhans MS, Niedrig M. 2012 The phylogeny of yellow fever virus 17D vaccines. Vaccine **30**, 989-994. (10.1016/j.vaccine.2011.12.057) 22197965

[7] Salmona M, Gazaignes S, Mercier-Delarue S, Garnier F, Korimbocus J, Colin de Verdière N, LeGoff J, Roques P, Simon F. 2015 Molecular characterization of the 17D-204 yellow fever vaccine. Vaccine **33**, 5432-5436. (10.1016/j.vaccine.2015.08.055) 26314624

[8] Camacho LAB, Freire M da S, Leal M da LF, de Aguiar SG, Nascimento JP do, Iguchi T, Lozana J de A, Farias RHG. 2004 Immunogenicity of WHO-17D and Brazilian 17DD yellow fever vaccines: a randomized trial. Rev. Saúde Pública **38**, 671-678. (10.1590/s0034-89102004000500009) 15499438

[9] Post PR, Santos CND, Carvalho R, Cruz ACR, Ricet CM, Galler R. 1992 Heterogeneity in envelope protein sequence and N-linked glycosylation among yellow fever virus vaccine strains. Virology **188**, 160-167. (10.1016/0042-6822(92)90745-b) 1566570

[10] Marchevsky RS, da Luz Leal M, Homma A, Coutinho ESF, Camacho LAB, Jabor AV, Galler R, Freire MS. 2006 Molecular and phenotypic analysis of a working seed lot of yellow fever virus 17DD vaccine strain produced from the secondary seed lot 102/84 with an additional passage in chicken embryos. Biologicals **34**, 191-197. (10.1016/j.biologicals.2005.09.005) 16326110

[11] Pestana CP, Lawson-Ferreira R, Lessa-Aquino C, Leal MDLF, Freire M da S, Homma A, Medeiros MA. 2018 Sanger-based sequencing technology for yellow fever vaccine genetic quality control. J. Virol. Methods **260**, 82-87. (10.1016/j.jviromet.2018.07.006) 30009851

[12] Beck AS, Barrett AD. 2015 Current status and future prospects of yellow fever vaccines. Expert Rev. Vaccines **14**, 1479-1492. (10.1586/14760584.2015.1083430) 26366673 PMC5563254

[13] Lauring AS, Andino R. 2010 Quasispecies theory and the behavior of RNA viruses. PLoS Pathog. **6**, e1001005. (10.1371/journal.ppat.1001005) 20661479 PMC2908548

[14] Vignuzzi M, Stone JK, Arnold JJ, Cameron CE, Andino R. 2005 Quasispecies diversity determines pathogenesis through cooperative interactions in a viral population. Nature **439**, 344-348. (10.1038/nature04388) 16327776 PMC1569948

[15] Beck AS, Wood TG, Widen SG, Thompson JK, Barrett ADT. 2018 Analysis by deep sequencing of discontinued neurotropic yellow fever vaccine strains. Sci. Rep. **8**, 13408. (10.1038/s41598-018-31085-2) 30194325 PMC6128858

[16] Ng S, Gisonni-Lex L, Azizi A. 2017 New approaches for characterization of the genetic stability of vaccine cell lines. Hum. Vaccines Immunother. **13**, 1669-1672. (10.1080/21645515.2017.1295191) PMC5512780 28333573

[17] Chumakov KM, Norwood LP, Parker ML, Dragunsky EM, Ran YX, Levenbook IS. 1992 RNA sequence variants in live poliovirus vaccine and their relation to neurovirulence. J. Virol. **66**, 966-970. (10.1128/jvi.66.2.966-970.1992) 1309923 PMC240798

[18] Galler R, Post PR, Santos CN, Ferreira II. 1998 Genetic variability among yellow fever virus 17D substrains. Vaccine **16**, 1024-1028. (10.1016/s0264-410x(97)00278-8) 9682354

[19] Sezerman OU, Ulgen E, Seymen N, Melis Durasi I. 2019 Bioinformatics workflows for genomic variant discovery, interpretation, and prioritization. In Bioinformatics tools for detection and clinical interpretation of genomic variations (eds A Samadikuchaksaraei, M Seifi), ch. 2. London, UK: IntechOpen. (10.5772/intechopen.85524)

[20] Depledge DP, Yamanishi K, Gomi Y, Gershon AA, Breuer J. 2016 Deep sequencing of distinct preparations of the live attenuated varicella-zoster virus vaccine reveals a conserved core of attenuating single-nucleotide polymorphisms. J. Virol. **90**, 8698-8704. (10.1128/jvi.00998-16) 27440875 PMC5021409

[21] Beck A, Tesh RB, Wood TG, Widen SG, Ryman KD, Barrett ADT. 2014 Comparison of the live attenuated yellow fever vaccine 17D-204 strain to its virulent parental strain asibi by deep sequencing. J. Infect. Dis. **209**, 334-344. (10.1093/infdis/jit546) 24141982 PMC3883177

[22] De Summa S, Malerba G, Pinto R, Mori A, Mijatovic V, Tommasi S. 2017 GATK hard filtering: tunable parameters to improve variant calling for next-generation sequencing targeted gene panel data. BMC Bioinf. **18**, 57-65. (10.1186/s12859-017-1537-8) PMC5374681 28361668

[23] Schneider T, Smith GH, Rossi MR, Hill CE, Zhang L. 2018 Validation of a customized bioinformatics pipeline for a clinical next-generation sequencing test targeting solid tumor-associated variants. J. Mol. Diagn. **20**, 355-365. (10.1016/j.jmoldx.2018.01.007) 29471113

[24] Posada-Cespedes S, Seifert D, Beerenwinkel N. 2017 Recent advances in inferring viral diversity from high-throughput sequencing data. Virus Res. **239**, 17-32. (10.1016/j.virusres.2016.09.016) 27693290

[25] Bolger AM, Lohse M, Usadel B. 2014 Trimmomatic: a flexible trimmer for Illumina sequence data. Bioinformatics **30**, 2114-2120. (10.1093/bioinformatics/btu170) 24695404 PMC4103590

[26] Bankevich A et al. 2012 SPAdes: a new genome assembly algorithm and its applications to single-cell sequencing. J. Comput. Biol. **19**, 455-477. (10.1089/cmb.2012.0021) 22506599 PMC3342519

[27] Robinson MD, Oshlack A. 2010 A scaling normalization method for differential expression analysis of RNA-seq data. Genome Biol. **11**, R25. (10.1186/gb-2010-11-3-r25) 20196867 PMC2864565

[28] Li H, Durbin R. 2010 Fast and accurate long-read alignment with Burrows-Wheeler transform. Bioinformatics **26**, 589-595. (10.1093/bioinformatics/btp698) 20080505 PMC2828108

[29] Li H et al. 2009 The sequence alignment/Map format and SAMtools. Bioinformatics **25**, 2078-2079. (10.1093/bioinformatics/btp352) 19505943 PMC2723002

[30] McKenna A et al. 2010 The genome analysis toolkit: a MapReduce framework for analyzing next-generation DNA sequencing data. Genome Res. **20**, 1297-1303. (10.1101/gr.107524.110) 20644199 PMC2928508

[31] Töpfer A, Zagordi O, Prabhakaran S, Roth V, Halperin E, Beerenwinkel N. 2013 Probabilistic inference of viral quasispecies subject to recombination. J. Comput. Biol. **20**, 113-123. (10.1089/cmb.2012.0232) 23383997 PMC3576916

[32] Bull RA et al. 2011 Sequential bottlenecks drive viral evolution in early acute hepatitis C virus infection. PLoS Pathog. **7**, e1002243. (10.1371/journal.ppat.1002243) 21912520 PMC3164670

[33] Abayasingam A et al. 2019 Genomic characterization of hepatitis C virus transmitted founder variants with deep sequencing. Infect. Genet. Evol. **71**, 36-41. (10.1016/j.meegid.2019.02.032) 30853512 PMC6487228

[34] Gouy M, Guindon S, Gascuel O. 2009 SeaView version 4: a multiplatform graphical user interface for sequence alignment and phylogenetic tree building. Mol. Biol. Evol. **27**, 221-224. (10.1093/molbev/msp259) 19854763

[35] Cacciabue M, Currá A, Carrillo E, König G, Gismondi MI. 2019 A beginner's guide for FMDV quasispecies analysis: sub-consensus variant detection and haplotype reconstruction using next-generation sequencing. Brief. Bioinform. **21**, 1766-1775. (10.1093/bib/bbz086) PMC7110011 31697321

[36] Li H, Homer N. 2010 A survey of sequence alignment algorithms for next-generation sequencing. Brief. Bioinform. **11**, 473-483. (10.1093/bib/bbq015) 20460430 PMC2943993

[37] Dimitrov KM et al. 2017 A robust and cost-effective approach to sequence and analyze complete genomes of small RNA viruses. Virol. J. **14**, 72. (10.1186/s12985-017-0741-5) 28388925 PMC5384157

[38] Eliseev A, Gibson KM, Avdeyev P, Novik D, Bendall ML, Pérez-Losada M, Alexeev N, Crandall KA. 2020 Evaluation of haplotype callers for next-generation sequencing of viruses. Infect. Genet. Evol. **82**, 104277. (10.1016/j.meegid.2020.104277) 32151775 PMC7293574

[39] Auwera GA et al. 2013 From FastQ data to high-confidence variant calls: the genome analysis toolkit best practices pipeline. Curr. Protoc. Bioinform. **43**, 11.10.1-11.10.33. (10.1002/0471250953.bi1110s43) PMC4243306 25431634

[40] Kum DB, Mishra N, Vrancken B, Thibaut HJ, Wilder-Smith A, Lemey P, Neyts J, Dallmeier K. 2019 Limited evolution of the yellow fever virus 17d in a mouse infection model. Emerg. Microbes Infect. **8**, 1734-1746. (10.1080/22221751.2019.1694394) 31797751 PMC6896426

[41] Davis EH, Beck AS, Strother AE, Thompson JK, Widen SG, Higgs S, Wood TG, Barrett ADT. 2019 Attenuation of live-attenuated yellow fever 17D vaccine virus is localized to a high-fidelity replication complex. mBio **10**, e02294-19. (10.1128/mbio.02294-19) 31641088 PMC6805994

